# Association between composite dietary antioxidant index and kidney stone prevalence in adults: data from National Health and Nutrition Examination Survey (NHANES, 2007–2018)

**DOI:** 10.3389/fnut.2024.1389714

**Published:** 2024-05-22

**Authors:** Qixin Duan, Han Huang, Shuang Zhang, Yang Wang, Dongming Lu, Lixin Wan, Yingming Sun, Yongyang Wu

**Affiliations:** ^1^Department of Urology, Nanyang Central Hospital, Nanyang, Henan, China; ^2^Department of Urology, Affiliated Sanming First Hospital of Fujian Medical University, Sanming, Fujian, China; ^3^Department of Nursing, Nanyang Central Hospital, Nanyang, Henan, China; ^4^Department of Oncology, Nanyang Central Hospital, Nanyang, Henan, China; ^5^Department of Medical and Radiation Oncology, Affiliated Sanming First Hospital of Fujian Medical University, Sanming, Fujian, China

**Keywords:** kidney stone, dietary antioxidants, composite dietary antioxidant index (CDAI), cross-sectional analysis, NHANES

## Abstract

**Background:**

The high prevalence of kidney stones in adults worldwide has prompted research into potential interventions, one of which involves exploring the consumption of antioxidants that may confer protective effects. However, the relationship between the composite dietary antioxidant index (CDAI), a crucial measure used to assess an individual’s overall antioxidant capacity from daily dietary intake, and kidney stones remains unclear. Therefore, we conducted cross-sectional analysis to examine the association between CDAI and kidney stone prevalence.

**Methods:**

The analysis was conducted utilizing data from the National Health and Nutrition Examination Survey (NHANES) from 2007 to 2018. Antioxidant intake was derived from two 24-h dietary recalls surveys, while CDAI, a comprehensive measure that includes antioxidants like vitamins A, C, and E, zinc, selenium, and carotenoids, was calculated. Multivariate logistic regression and restricted cubic spline (RCS) regression were utilized to examine the association between CDAI and the prevalence of kidney stones.

**Results:**

The study included a total of 28,516 participants, with 2,748 individuals having a history of kidney stones. The median of CDAI was −0.01 (−2.02, 2.37). Individuals in the fourth quartile of CDAI exhibited a significantly lower prevalence of kidney stones compared to those in the first quartile (Odds Ratio [OR] = 0.769 [0.633–0.935]), even after adjusting for potential confounding factors (including age, sex, race, education level, poverty income ratio, smoking status, drinking status, body mass index (BMI), energy intake levels, physical activity level, serum calcium concentration, estimated glomerular filtration rate (eGFR), hypertension, diabetes and supplement use). The RCS analysis revealed a non-linear relationship between CDAI and kidney stone prevalence, with inflection points identified at 0.06 (*p* for non-linearity = 0.039). Subgroup analysis demonstrated consistent CDAI-kidney stone prevalence associations across all subsets. Furthermore, a significant inverse correlation was observed between CDAI and inflammatory markers.

**Conclusion:**

This study provides evidence supporting a reciprocal correlation between adult dietary antioxidant intake, as measured by CDAI, and kidney stone prevalence. These findings emphasize the potential benefits of consuming dietary antioxidants in lowering the risk of kidney stone formation.

## 1 Introduction

Formation of kidney stones results from the abnormal accumulation of crystalline substances, including calcium, oxalic acid, uric acid, and cystine, within the renal calyces and pelvis system (1). Over the past few years, there has been a notable rise in the occurrence of kidney stones, presenting a major issue for public health (2). It is estimated that approximately 11% of the population in the United States will experience kidney stones during their lifetimes (3). These stones cause severe pain and discomfort, significantly impacting the quality of life for individuals affected (4). Furthermore, kidney stones can result in complications such as urinary flow obstruction, urinary tract infections, and in some cases, even renal damage (5, 6).The process is intricate and multifaceted, with dietary factors, particularly food choices, implicated in their formation (7). Notably, extensive attention has been dedicated to investigating the potential protective properties exhibited by dietary antioxidant compounds against the development of kidney stones (8).

Various approaches, including lifestyle modification, physical activity, and particularly nutritional interventions, have been proposed in numerous studies for the prevention and treatment of various chronic diseases such as renal dysfunction (9–11). Antioxidants are essential for neutralizing the harmful impacts of reactive oxygen species (ROS) produced in normal cellular activities (12). Imbalance between ROS generation and the body’s antioxidant capacity has been associated with various clinical conditions, including kidney stone formation (13). By reducing ROS levels, antioxidants have the potential to protect tubular cells from damage, thereby impeding mineral and crystal accumulation and ultimately preventing kidney stone formation (14). Dietary antioxidant compounds, such as β-carotene and β-cryptoxanthin, hold promise for the prevention of kidney stones (8). However, there is a significant association between total and supplemental vitamin C intake and an increased risk of kidney stones in men (15). Some dietary antioxidants, including retinol, β-carotene, vitamins B6, C, and E, and lycopene, were not observed to be associated with kidney stone risk in another study (16). These inconsistent outcomes regarding individual dietary antioxidants necessitate further investigations or comprehensive indicators to establish a definitive correlation between dietary antioxidants and susceptibility to kidney stones.

In this context, the Composite Dietary Antioxidant Index (CDAI) plays a significant role as a comprehensive measure of total antioxidant consumption (17). By encompassing both micronutrients (e.g., vitamins A, C, and E) and non-vitamin antioxidants (e.g., zinc, selenium and carotenoids), the CDAI measures antioxidant consumption from various food sources and consolidates them into a single score. Adequate intake of antioxidants, as measured by CDAI, has been linked to a lower likelihood of developing chronic illnesses like heart disease, cancer, hypertension, osteoarthritis and osteoporosis (18–22). Nevertheless, there is no research that has proven a connection between CDAI and the occurrence of kidney stones.

To explore the correlation between CDAIs and the occurrence of kidney stones in a diverse population, we carefully examined dietary antioxidant data from the 2007–2018 National Health and Nutrition Examination Survey (NHANES). Through exhaustive and rigorous analysis, our aim is to provide valuable insights into the role of dietary antioxidants in kidney stone pathogenesis and potentially discover innovative strategies for prevention and management of this condition.

## 2 Materials and methods

### 2.1 Study population

The NHANES initiative, overseen by the Centers for Disease Control and Prevention (CDC) in the United States, aims to assess the health, diet, and general welfare of individuals (23). NHANES collects data from a nationally representative sample of individuals through surveys, medical examinations, and laboratory tests. This comprehensive survey covers various domains, including chronic and infectious diseases, obesity, diabetes, cardiovascular health, nutrition, environmental exposures, and oral health. Approval for the research protocols was granted by the National Center for Health Statistics (NCHS) Research Ethics Review Board, with all participants giving informed consent.

**Figure 1:**
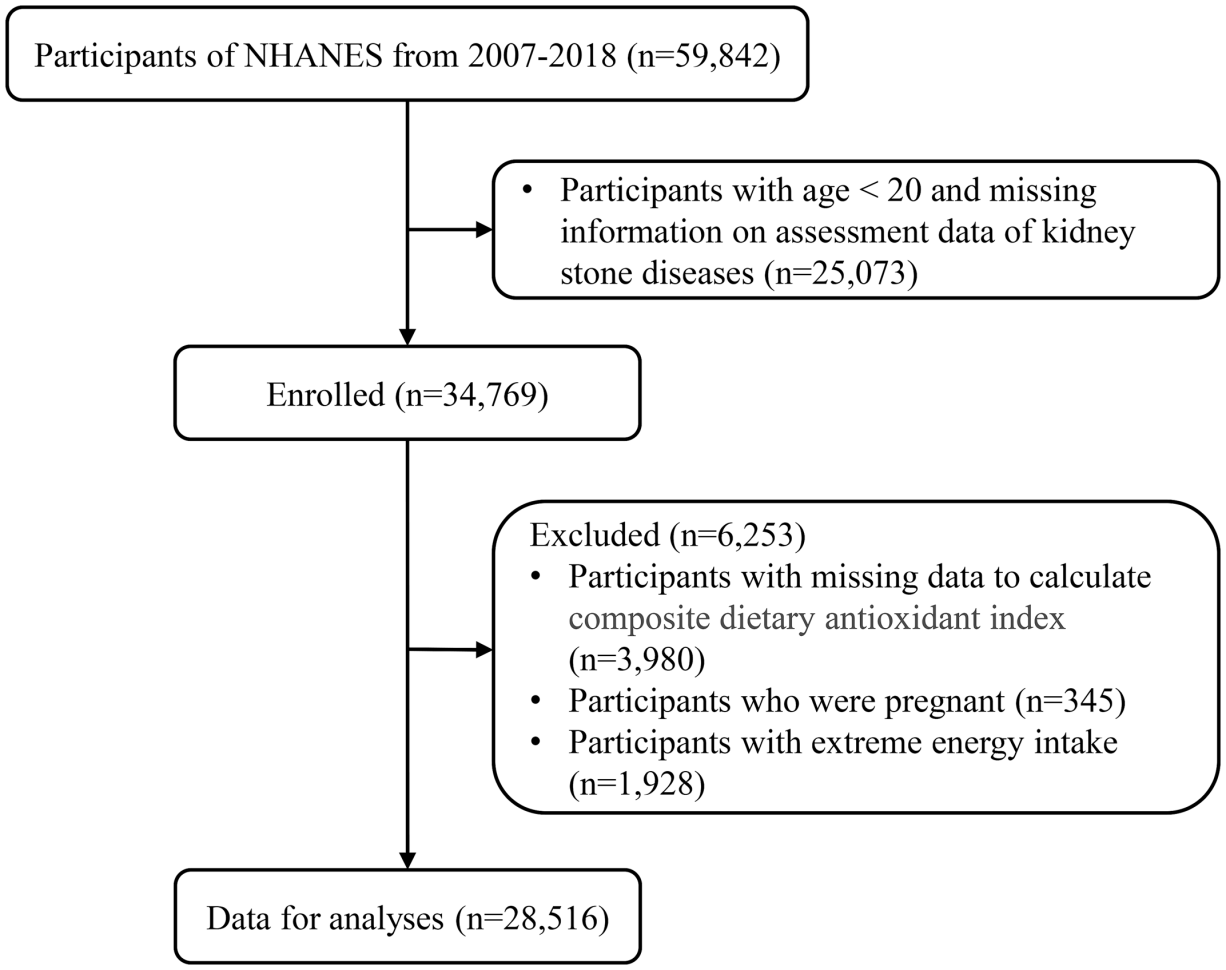
Flowchart of the study.

In our research, a grand total of 59,842 people took part in the NHANES project from 2007 to 2018. We excluded 25,073 individuals below the age of 20 and those lacking information on kidney stones. Additionally, 3,980 individuals without the necessary data for calculating CDAI, 345 pregnant individuals, and 1,928 individuals with extreme energy intakes were also excluded. As a result, our analysis included a final sample size of 28,516 participants (Figure 1).

### 2.2 Assessment of CDAI and kidney stones

The CDAI considers the consumption of diet antioxidants, including vitamins A, C, and E, zinc, selenium, and carotenoids. To calculate the CDAI, we exercised a modified version of the CDAI protocol developed by Wright et al. (24). In short, the standardized values for each antioxidant were obtained by subtracting mean and dividing by their standard deviation. Then, we calculated the CDAI by adding standardized values of all six antioxidants. A greater rating signifies a higher consumption of diet antioxidants. The average of two 24-h recall interviews was used to collect information on dietary antioxidants. To ensure data accuracy and reliability, individuals with abnormal total energy intake (>4,200 or < 800 kcal/day in males; >3,500 or < 500 kcal/day in females) were excluded (25). Participants were categorized as having kidney stones if they affirming the following question: Has a doctor or other health professional ever told you that you have kidney stones? (26).

$\mathrm{CDAI}=\overset{n=6}{\underset{i=1}{\sum }}\;\left(\mathrm{Individual}\;\mathrm{Intake}-\mathrm{Mean}\right)/SD$

### 2.3 Covariates

To mitigate potential confounding bias in our analyses, we selected covariates based on clinical expertise and previous research (27). The NHANES datasets provided information, including age (years), sex (male or female), race/ethnicity (Mexican American, other Hispanic, non-Hispanic White, non-Hispanic Black, or other), education level (below high school, high school, or above high school), total energy intake (kcal/day), serum calcium (mmol/L), estimated glomerular filtration rate (eGFR; ml/min/1.73 m2), and supplement use (%). Poverty Income Ratio (PIR) was determined by dividing an individual’s income by the poverty threshold and then classified into one of three brackets (1.0, 1.1–3.0, or > 3.0) (28). Smoking status was categorized as never smokers (<100 cigarettes), current smokers (>100 cigarettes), and former smokers (>100 cigarettes and having quit smoking) (29). Participants were categorized based on their drinking habits as nondrinkers, low-to-moderate drinkers (men <2 drinks/day; women <1 drinks/day), or heavy drinkers (29). Physical activity was classified as inactive (no leisure-time physical activity), insufficiently active (moderate activity 1–5 times per week with metabolic equivalents [MET] 3–6 or vigorous activity 1–3 times per week with MET >6), and active (individuals engaging in more moderate or vigorous activity than mentioned above) (30). Body mass index (BMI) was used to assess weight status, categorizing individuals as normal weight (<25.0 kg/m2), overweight(25.0–29.9 kg/m2), or obese (>29.9 kg/m2) (31). The frequency of hypertension and diabetes was determined based on self-reported questionnaires.

### 2.4 Statistical analysis

Baseline characteristics were presented as means with standard errors (SEs), medians with interquartile ranges (IQRs), or numbers with percentages. The Student’s t-test or Mann–Whitney U test were used to compare continuous variables, while the chi-square test was used to compare categorical variables. The relationship between CDAI and kidney stones prevalence was explored using multifactorial logistic regression. The potential nonlinear association was assessed using a restricted cubic spline (RCS) model with three knots placed at the 10th, 50th, and 90th percentiles of CDAI (32, 33). The median was set as the reference value. Additionally, stratified analyses were conducted to investigate factors that might influence the connection between CDAI and kidney stone prevalence. Finally, the multiple linear regression relationship between CDAI quartiles and inflammation indicators was determined. R (version 4.2.0) was used for all analyses, with statistical significance defined as *p*-values below 0.05. To ensure reliable national estimates, data analysis considered the primary sampling units, sample weights, and strata, following the guidelines provided by the National Center for Health Statistics.

## 3 Results

### 3.1 Baseline characteristics of the participants

The characteristics of participants in the 2007–2018 NHANES are detailed in Table 1. This research included 28,516 people, 2,748 of whom had previous experience of kidney stones. The sample consisted of 48.03% male individuals and 42.04% non-Hispanic White participants. Significant differences were observed in all baseline variables when comparing individuals with and without kidney stones, except for education level, total energy intake, and serum calcium (*p* > 0.05). Patients with kidney stones were higher proportions of older males, non-Hispanic white participants, former smokers, low-to-moderate drinkers, and physically inactive individuals (*p* < 0.01). Additionally, they had a higher prevalence of metabolic disorders including obesity, hypertension and diabetes (*p* < 0.01). We performed an analysis of baseline characteristics of the general adult population based on CDAI quartiles (Supplementary Table 1). Participants in the highest quartile of the CDAI were more likely to be females, better educated adults, better off families, nonsmokers, low-to-moderate drinkers, normal weight and physically active individuals, as well as non-hypertensive and non-diabetic individuals (*p* < 0.05).

**Table 1:** Baseline characteristics of the general adult population in NHANES 2007–2018.

Characteristics	Total (*n* = 28,516)	Kidney stones		*p* value
		No (*n* = 25,768)	Yes (*n* = 2,748)	
Age, years	48.06 (0.24)	47.39 (0.25)	54.06 (0.34)	<0.001
Sex, %				<0.001
Female	14,820 (51.97)	13,589 (53.17)	1,231 (45.98)	
Male	13,696 (48.03)	12,179 (46.83)	1,517 (54.02)	
Race/ethnicity, %				<0.001
Mexican American	4,252 (14.91)	3,900 (8.54)	352 (5.87)	
Other Hispanic	2,965 (10.4)	2,652 (5.75)	313 (5.09)	
Non-Hispanic White	11,988 (42.04)	10,479 (66.38)	1,509 (77.37)	
Non-Hispanic Black	6,018 (21.1)	5,664 (11.44)	354 (5.61)	
Other race	3,293 (11.55)	3,073 (7.88)	220 (6.06)	
Education level, %				0.950
Below high school	6,859 (24.05)	6,178 (15.32)	681 (15.37)	
High school	6,500 (22.79)	5,881 (23.02)	619 (22.74)	
Above high school	15,157 (53.15)	13,709 (61.66)	1,448 (61.89)	
Family PIR, %				0.020
≤1.0	6,120 (21.46)	5,562 (14.61)	558 (12.20)	
1.1–3.0	12,029 (42.18)	10,841 (35.83)	1,188 (36.32)	
>3.0	10,367 (36.36)	9,365 (49.56)	1,002 (51.48)	
Smoking status, %				<0.001
Never smoker	15,953 (55.94)	14,602 (56.80)	1,351 (50.57)	
Former smoker	6,953 (24.38)	6,096 (24.34)	857 (30.43)	
Current smoker	5,610 (19.67)	5,070 (18.86)	540 (19.00)	
Drinking status, %				0.001
Nondrinker	6,516 (22.85)	5,852 (17.84)	664 (19.87)	
Low-to-moderate drinker	19,795 (69.42)	17,867 (72.61)	1928 (73.34)	
Heavy drinker	2,205 (7.73)	2049 (9.55)	156 (6.78)	
Body mass index, %				<0.001
<25.0 kg/m2	7,986 (28.01)	7,464 (30.20)	522 (19.36)	
25.0–29.9 kg/m2	9,403 (32.97)	8,467 (32.93)	936 (32.59)	
>29.9 kg/m2	11,127 (39.02)	9,837 (36.87)	1,290 (48.06)	
Physical activity, %				<0.001
Inactive	7,670 (26.9)	6,769 (21.67)	901 (28.03)	
Insufficiently active	8,943 (31.36)	8,133 (32.37)	810 (30.07)	
Active	11,903 (41.74)	10,866 (45.96)	1,037 (41.90)	
Total energy intakes, kcal/day				0.370
Quartile 1	7,135 (25.02)	6,443 (21.82)	692 (21.47)	
Quartile 2	7,134 (25.02)	6,430 (24.42)	704 (24.84)	
Quartile 3	7,120 (24.97)	6,424 (25.85)	696 (27.62)	
Quartile 4	7,127 (24.99)	6,471 (27.91)	656 (26.06)	
Serum calcium, mmol/L	2.35 (0.00)	2.35 (0.00)	2.34 (0.00)	0.070
eGFR, ml/min/1.73 m2	93.83 (0.32)	94.58 (0.33)	87.13 (0.50)	<0.001
Self-reported hypertension, %				<0.001
No	17,932 (62.88)	16,588 (69.08)	1,344 (52.54)	
Yes	10,584 (37.12)	9,180 (30.92)	1,404 (47.46)	
Self-reported diabetes, %				<0.001
No	24,625 (86.36)	22,512 (90.87)	2,113 (81.02)	
Yes	3,891 (13.64)	3,256 (9.13)	635 (18.98)	
Supplement use, %				0.010
No	13,977 (49.01)	12,729 (46.16)	1,248 (42.91)	
Yes	14,539 (50.99)	13,039 (53.84)	1,500 (57.09)	
CDAI	0.55 (0.05)	0.59 (0.05)	0.26 (0.10)	0.002

PIR, poverty income ratio; eGFR, estimated glomerular filtration rate; CDAI, composite dietary antioxidant index. Normally distributed continuous variables are described as means ± SEs, and continuous variables without a normal distribution are presented as medians [interquartile ranges]. Sampling weights were applied for calculation of demographic descriptive statistics; N reflect the study sample while percentages reflect the survey-weighted data.

### 3.2 Distribution and concentration of CDAI in adults individuals with kidney stones

The distribution and concentration of CDAI in adult patients with kidney stones are shown in Table 2, and the median of CDAI was −0.01(−2.02, 2.37). In this study participants, the average daily consumption of vitamins A, C, and E, zinc, selenium, and carotenoids was 490 (276, 490) μg/day, 50.7 (20.90, 109.40) mg/day, 7.00 (4.57, 10.48) mg/day, 9.84 (6.87, 13.92) mg/day, 101.30 (71.40, 139.00) μg/day, and 5335.00 (2049.00, 12025.00) μg/day, respectively.

**Table 2:** Distributions and concentrations of composite dietary antioxidant index (CDAI) among adults in NHANES 2007–2018.

	Mean	5th	25th	50th	75th	95th
CDAI	0.55	−4.23	−2.02	−0.01	2.37	7.17
Vitamins A, μg/day	620.63	89.00	276.00	490.00	796.00	1525.00
Vitamins C, mg/day	79.19	4.30	20.90	50.70	109.40	241.30
Vitamins E, mg/day	8.44	2.19	4.57	7.00	10.48	19.21
Zinc, mg/day	11.14	3.81	6.87	9.84	13.92	22.15
Selenium, μg/day	110.47	39.50	71.40	101.30	139.00	209.40
Carotenoid, μg/day	9299.45	341.00	2049.00	5335.00	12025.00	31193.00

5^th^, 5th percentile; 25^th^, 25th percentile; 50^th^, 50th percentile; 75^th^, 75th percentile; 95^th^, 95th percentile.

### 3.3 Association between CDAI and kidney stones

**Figure 2:**
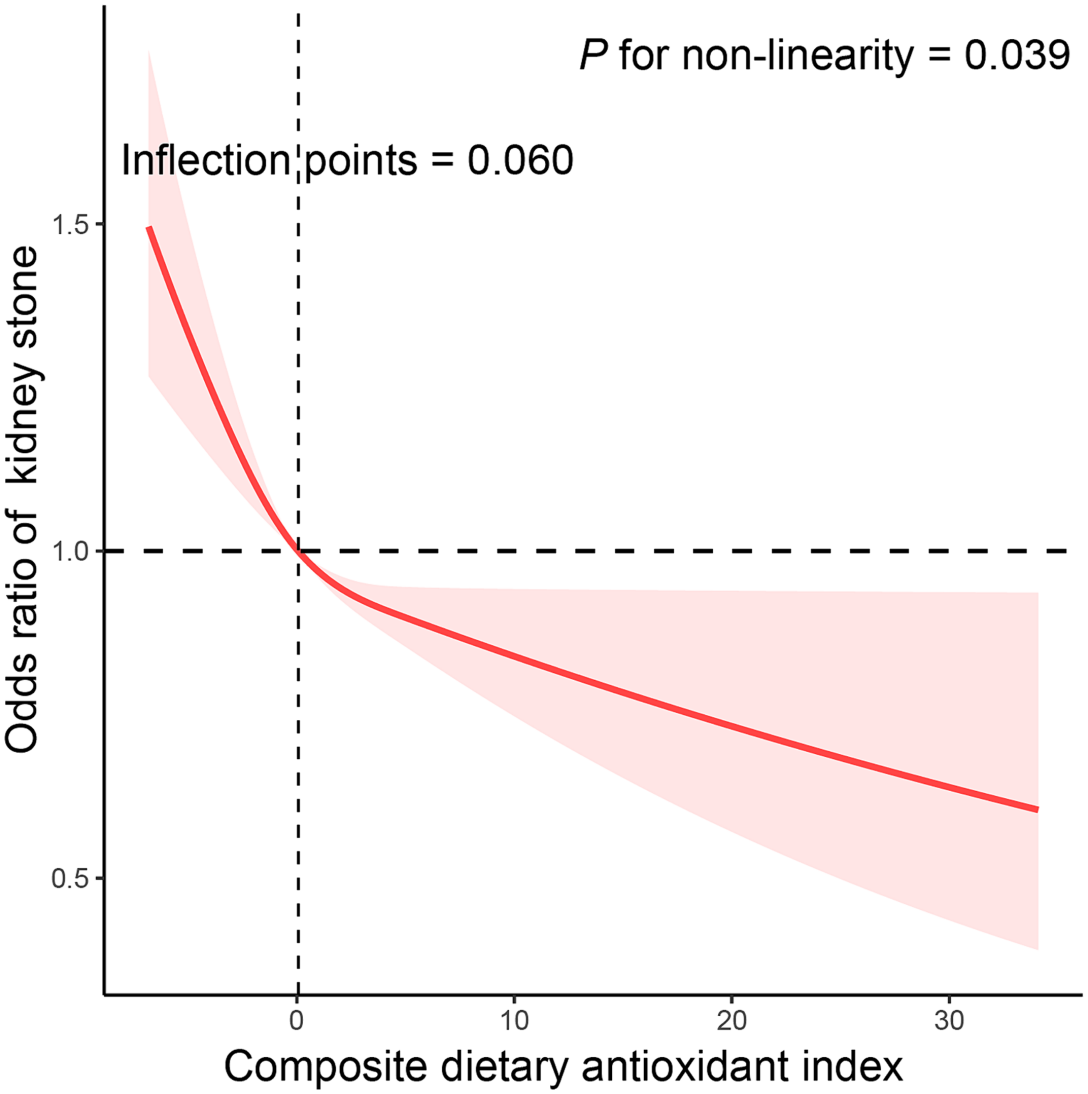
The exposure-response association of the composite dietary antioxidant index (CDAI) with the prevalence of kidney stone by restricted cubic spline (RCS). Model was adjusted for age (continuous), sex (male or female), race (Mexican American, Other Hispanic, Non-Hispanic White, Non-Hispanic Black or Other), education level (below high school, high school, or above high school), family income-to-poverty ratio (≤1.0, 1.1–3.0, or > 3.0), smoking status (never smoker, former smoker, or current smoker), drinking status (nondrinker, low-to-moderate drinker, or heavy drinker), BMI (<25.0, 25.0–29.9, or > 29.9), energy intake levels (in quartiles), physical activity (inactive, insufficiently active, or active), serum calcium (continuous), eGFR (continuous), hypertension (yes or no), diabetes (yes or no), and supplement use (yes or no).

We utilized CDAI as a continuous variable and four categorical factors to explore the relationship between CDAI and kidney stone prevalence. By analysis of the continuous independent variable, higher continuous CDAI was substantially linked with reduced kidney stone prevalence in all models (Table 3). In the crude model, a statistically significant inverse correlation between CDAI and kidney stone prevalence was reported in the categorical independent variable analysis. This connection remained even after additional modifications for age, sex, and ethnicity. In model 2, it was discovered that people in the highest quarter of CDAI had a 23.1% reduced likelihood of kidney stones compared to those in the lowest quarter (OR = 0.769 [0.633–0.935], *p*_trend_ = 0.003). The results were similar even when subjects with cancer, thyroid disease, fatty liver, stroke, end-stage renal disease, and taking corticosteroid medications were excluded (Supplementary Tables 2–4). Additionally, the correlation between CDAI and kidney stones was examined in more detail through the use of RCS (Figure 2). A non-linear and negative correlation was found between CDAI and the occurrence of kidney stones, with inflection points at 0.06 (*p* for non-linearity = 0.039).

**Table 3:** ORs (95% CIs) of the prevalence of kidney stone according to quartiles of composite dietary antioxidant index (CDAI) among adults in NHANES 2007–2018.

	Crude	Model 1	Model 2
	OR (95% CI)	OR (95% CI)	OR (95% CI)
Continuous CDAI	0.976 (0.960–0.992)	0.973 (0.956–0.990)	0.973 (0.953–0.993)
P value	0.005	0.002	0.009
Quartiles of CDAI			
Quartile 1	1 [Reference]	1 [Reference]	1 [Reference]
Quartile 2	1.023 (0.872–1.200)	0.964 (0.815–1.140)	0.929 (0.774–1.116)
Quartile 3	0.899 (0.757–1.068)	0.849 (0.710–1.014)	0.825 (0.681–0.999)
Quartile 4	0.831 (0.701–0.986)	0.787 (0.658–0.941)	0.769 (0.633–0.935)
P for trend	0.009	0.003	0.003

OR, odds ratio; CI, confidence interval; Model 1 was adjusted for age (continuous), sex (male or female), and race (Mexican American, Other Hispanic, Non-Hispanic White, Non-Hispanic Black or Other); Model 2 was adjusted for Model 1 plus education level (below high school, high school, or above high school), family income-to-poverty ratio (≤1.0, 1.1–3.0, or > 3.0), smoking status (never smoker, former smoker, or current smoker), drinking status (nondrinker, low-to-moderate drinker, or heavy drinker), BMI (<25.0, 25.0–29.9, or > 29.9), energy intake levels (in quartiles), physical activity (inactive, insufficiently active, or active), serum calcium (continuous), eGFR (continuous), hypertension (yes or no), diabetes (yes or no), and supplement use (yes or no).

### 3.4 Association between the components of CDAI and kidney stones

The correlation between individual elements of the CDAI and occurrence of kidney stones was analyzed (Table 4). Lower prevalence of kidney stones was linked to the fourth quartile of vitamin C (OR = 0.777 [0.648–0.932], *p*_trend_ = 0.008), zinc (OR = 0.841 [0.712–0.992], *p*_trend_ = 0.006), and selenium (OR = 0.772 [0.652–0.914], *p*_trend_ = 0.002). RCS was used to explore the dose–response association between each antioxidant micronutrient and kidney stones (Supplementary Figure 1).

**Table 4:** ORs (95% CIs) of the prevalence of kidney stone according to quartiles of dietary antioxidant micronutrients among adults in NHANES 2007–2018.

	Quartiles of dietary antioxidant micronutrients				
	Quartile 1	Quartile 2	Quartile 3	Quartile 4	*p* trend
Vitamins A	1 [Reference]	0.939 (0.777–1.133)	0.917 (0.744–1.131)	0.920 (0.761–1.113)	0.444
Vitamins C	1 [Reference]	0.925(0.782–1.094)	0.878 (0.723–1.066)	0.777 (0.648–0.932)	0.008
Vitamins E	1 [Reference]	1.069 (0.906–1.261)	0.857 (0.712–1.032)	0.994 (0.843–1.172)	0.740
Zinc	1 [Reference]	1.090 (0.956–1.243)	0.913 (0.783–1.065)	0.841 (0.712–0.992)	0.006
Selenium	1 [Reference]	0.940 (0.822–1.075)	0.877 (0.757–1.016)	0.772 (0.652–0.914)	0.002
Carotenoid	1 [Reference]	0.928 (0.777–1.109)	0.941 (0.789–1.122)	0.852 (0.696–1.044)	0.142

OR, odds ratio; CI, confidence interval; Model was adjusted for age (continuous), sex (male or female), race (Mexican American, Other Hispanic, Non-Hispanic White, Non-Hispanic Black or Other), education level (below high school, high school, or above high school), family income-to-poverty ratio (≤1.0, 1.1–3.0, or > 3.0), smoking status (never smoker, former smoker, or current smoker), drinking status (nondrinker, low-to-moderate drinker, or heavy drinker), BMI (<25.0, 25.0–29.9, or > 29.9), energy intake levels (in quartiles), physical activity (inactive, insufficiently active, or active), serum calcium (continuous), eGFR (continuous), hypertension (yes or no), diabetes (yes or no), and supplement use (yes or no).

### 3.5 Stratified analysis

Subgroup analyses were performed according to age, gender, ethnicity, smoking and alcohol habits, level of physical activity, body mass index, use of supplements, and eGFR (Table 5). RCS was employed to assess the concordance between CDAI and the risk of kidney stones in stratifying alcohol consumption and age (Supplementary Figure 2). Consistency in the relationship between CDAI and incidence of kidney stones was observed in all subcategories. The initial model was adjusted for total water intake, dietary calcium intake, serum phosphate and vitamin D levels, as well as various dietary factors and nutrient biomarkers. Notably, the final regression analysis yielded consistent results without significant changes, reaffirming the robustness of our findings (Table 6).

**Table 5:** Stratified analyses of the associations between quartiles of composite dietary antioxidant index (CDAI) and the prevalence of kidney stone in NHANES 2007–2018.

Subgroups	N	Quartiles of CDAI				*p*-interaction
		Quartile 1	Quartile 2	Quartile 3	Quartile 4	
Age, years						
20–39	9,102	1 [Reference]	0.881(0.642–1.210)	0.677(0.463–0.989)	0.760(0.529–1.090)	0.067
40–59	9,434	1 [Reference]	0.937(0.689–1.274)	0.729(0.531–0.999)	0.638(0.448–0.908)	
≥ 60	9,980	1 [Reference]	0.993(0.765–1.289)	1.088(0.802–1.477)	0.994(0.713–1.386)	
Sex, %						
Female	14,820	1 [Reference]	0.952(0.733–1.237)	0.727(0.561–0.943)	0.787(0.582–1.063)	0.252
Male	13,696	1 [Reference]	0.920(0.725–1.167)	0.915(0.701–1.195)	0.767(0.575–1.022)	
Race, %						
Non-Hispanic White	11,988	1 [Reference]	0.679(0.515–0.895)	0.623(0.447–0.869)	0.727(0.490–1.079)	0.125
Non-Hispanic Black	6,018	1 [Reference]	1.033(0.824–1.295)	0.908(0.708–1.165)	0.801(0.617–1.041)	
Other	10,510	1 [Reference]	0.736(0.526–1.029)	0.684(0.491–0.952)	0.762(0.502–1.156)	
Smoking status, %						
Never smoker	15,953	1 [Reference]	0.936(0.720–1.216)	0.835(0.620–1.124)	0.721(0.538–0.967)	
Former smoker	6,953	1 [Reference]	0.954(0.705–1.290)	0.839(0.623–1.130)	0.854(0.622–1.174)	0.993
Current smoker	5,610	1 [Reference]	0.921(0.585–1.449)	0.818(0.568–1.178)	0.830(0.518–1.330)	
Drinking status, %						
Nondrinker	6,516	1 [Reference]	1.137(0.811–1.594)	0.976(0.692–1.375)	1.018(0.657–1.578)	
Low-to-moderate drinker	19,795	1 [Reference]	0.886(0.734–1.070)	0.775(0.634–0.949)	0.693(0.564–0.852)	0.388
Heavy drinker	2,205	1 [Reference]	0.762(0.381–1.523)	0.897(0.455–1.770)	0.910(0.456–1.815)	
Physical activity, %						
Inactive	7,670	1 [Reference]	1.036(0.800–1.340)	0.895(0.655–1.223)	0.900(0.658–1.231)	
Insufficiently active	8,943	1 [Reference]	0.807(0.597–1.092)	0.816(0.596–1.119)	0.722(0.487–1.072)	0.625
Active	11,903	1 [Reference]	0.952(0.729–1.243)	0.777(0.574–1.053)	0.737(0.552–0.984)	
Body mass index, %						
<25.0 kg/m2	7,986	1 [Reference]	0.982(0.671–1.437)	1.190(0.769–1.843)	1.026(0.670–1.572)	
25.0–29.9 kg/m2	9,403	1 [Reference]	1.098(0.835–1.444)	0.871(0.634–1.196)	0.735(0.517–1.045)	0.328
>29.9 kg/m2	11,127	1 [Reference]	0.806(0.623–1.043)	0.673(0.520–0.871)	0.714(0.561–0.910)	
Supplement use, %						
No	13,977	1 [Reference]	0.937(0.724–1.213)	0.801(0.626–1.025)	0.712(0.548–0.925)	0.799
Yes	14,539	1 [Reference]	0.915(0.719–1.164)	0.848(0.647–1.113)	0.810(0.630–1.042)	
eGFR, ml/min/1.73 m2						
<90	11,737	1 [Reference]	0.971(0.773–1.219)	0.897(0.679–1.184)	0.872(0.642–1.184)	0.452
≥ 90	16,779	1 [Reference]	0.898(0.676–1.193)	0.762(0.576–1.007)	0.674(0.511–0.888)	

Analyses were adjusted for covariates age (continuous), sex (male or female), race (Mexican American, Other Hispanic, Non-Hispanic White, Non-Hispanic Black or Other), education level (below high school, high school, or above high school), family income-to-poverty ratio (≤1.0, 1.1–3.0, or > 3.0), smoking status (never smoker, former smoker, or current smoker), drinking status (nondrinker, low-to-moderate drinker, or heavy drinker), BMI (<25.0, 25.0–29.9, or > 29.9), energy intake levels (in quartiles), physical activity (inactive, insufficiently active, or active), serum calcium (continuous), eGFR (continuous), hypertension (yes or no), diabetes (yes or no), and supplement use (yes or no) when they were not the strata variables.

**Table 6:** ORs (95% CIs) of the prevalence of kidney stone according to quartiles of composite dietary antioxidant index (CDAI) among adults with further adjustment of dietary factors and nutrient biomarkers in NHANES 2007–2018.

	Quartiles of CDAI				*p* trend
	Quartile 1	Quartile 2	Quartile 3	Quartile 4	
Model + total water intake	1 [Reference]	0.93 (0.77–1.11)	0.82 (0.68–1.00)	0.76 (0.63–0.92)	0.002
Model + dietary calcium intake	1 [Reference]	0.94 (0.79–1.13)	0.85 (0.70–1.04)	0.81 (0.66–0.99)	0.019
Model + dietary vitamin D intake	1 [Reference]	0.96 (0.80–1.15)	0.86 (0.71–1.05)	0.82 (0.66–0.99)	0.023
Model + serum phosphate	1 [Reference]	0.93 (0.78–1.12)	0.84 (0.69–1.01)	0.78 (0.64–0.95)	0.004
Model + serum vitamin D	1 [Reference]	0.93 (0.77–1.12)	0.82 (0.68–1.00)	0.77 (0.63–0.94)	0.003
Model + dietary factors*	1 [Reference]	0.96 (0.80–1.14)	0.87 (0.71–1.05)	0.82 (0.67–0.99)	0.029
Model + nutrient biomarkers†	1 [Reference]	0.93 (0.78–1.12)	0.83 (0.69–1.01)	0.77 (0.64–0.94)	0.003

Model was adjusted for age (continuous), sex (male or female), race (Mexican American, Other Hispanic, Non-Hispanic White, Non-Hispanic Black or Other), education level (below high school, high school, or above high school), family income-to-poverty ratio (≤1.0, 1.1–3.0, or > 3.0), smoking status (never smoker, former smoker, or current smoker), drinking status (nondrinker, low-to-moderate drinker, or heavy drinker), BMI (<25.0, 25.0–29.9, or > 29.9), energy intake levels (in quartiles), physical activity (inactive, insufficiently active, or active), serum calcium (continuous), eGFR (continuous), hypertension (yes or no), diabetes (yes or no), and supplement use (yes or no). *Further adjusted for Model + total water intake, and dietary factors (dietary calcium intake [in quartiles], and dietary vitamin D intake [in quartiles]). †Further adjusted for Model + total water intake, and nutrient biomarkers (serum phosphate [continuous] and serum vitamin D [in quartiles]).

### 3.6 Association between CDAI and inflammatory markers

In addition, we examined the relationship between CDAI quartiles and inflammatory markers (Table 7). Interestingly, a significant negative correlation was observed between a high CDAI quartile and inflammatory markers (such as alkaline phosphatase [ALP], white blood cell count [WBC], neutrophil count [NEU], monocyte count [MON], and red blood cell distribution width [RDW]; *p* < 0.05).

**Table 7:** Multiple linear regression associations of quartiles of composite dietary antioxidant index (CDAI) with inflammatory markers in adults.

	Quartiles of CDAI				
	*β*	*β* (95% CI)	*β* (95% CI)	*β* (95% CI)	*p* trend
ALP	0 [Reference]	−0.024 (−0.039, −0.010)	−0.040 (−0.053, −0.026)	−0.051 (−0.067, −0.034)	<0.001
WBC	0 [Reference]	−0.004 (−0.018, 0.009)	−0.016 (−0.031, −0.001)	−0.024 (−0.040, −0.009)	0.002
NEU	0 [Reference]	−0.010 (−0.028, 0.008)	−0.022 (−0.042, −0.001)	−0.027 (−0.048, −0.007)	0.008
MON	0 [Reference]	0.001 (−0.015, 0.017)	−0.011 (−0.027, 0.004)	−0.016 (−0.033, 0.002)	0.039
RDW	0 [Reference]	−0.085 (−0.140, −0.031)	−0.114 (−0.178, −0.049)	−0.136 (−0.194, −0.078)	<0.001

CI, confidence interval; ALP, alkaline phosphatase; WBC, white blood cell count; NEU, neutrophil count; MON, monocyte count; RDW, red blood Cell distribution width.

## 4 Discussion

In this cross-sectional study, we included 28,516 individuals to analyze the potential link between CDAI and kidney stone prevalence. After controlling for all confounding factors, an inverse correlation between CDAI and kidney stone prevalence was discovered. A decrease in the occurrence of kidney stones was associated with the highest quartile of CDAI (OR = 0.769 [0.633–0.935], *p*_trend_ = 0.003). We discovered a non-linear and negative relationship between CDAI and kidney stone, with inflection points of 0.06. Furthermore, the stratified analysis did not reveal any variables that had a significant impact on the outcomes.

The CDAI is a novel index that quantifies the overall dietary antioxidant capacity based on the consumption of various antioxidants (17). Several studies have investigated the relation between CDAI and conditions such as hypertension, cancer, and depression (19, 21, 34). These studies indicate that increased CDAI scores are linked to a lower likelihood of specific diseases. For example, a study discovered that a higher CDAI score was associated with a reduced risk of mortality due to cardiovascular disease (18). Another study revealed that individuals with elevated CDAI scores had a decreased risk of developing colorectal cancer (35). This pilot investigation aims to explore the relationship between CDAI and kidney stone. The results of our study show a strong association between elevated CDAI scores and a reduced incidence of kidney stones, even after accounting for all potential influencing factors.

Dietary antioxidants are compounds designed to shield cells and tissues from the detrimental impacts of free radicals (36). A growing body of evidence indicates a link between consuming antioxidants and reducing the risk of chronic illnesses like heart disease, stroke, Alzheimer’s, and cancer (37–40). Antioxidants can minimize the risk of certain diseases by neutralizing free radicals, which are recognized for causing damage to blood vessels, DNA, and other cellular components (41). Studies have investigated the association between individual antioxidant micronutrients and kidney stones. Selenium, an essential trace element for the human body, enhances antioxidative capacity, scavenges free radicals, reduces oxidative damage caused by reactive oxygen species to the kidneys, and inhibits oxalate synthesis (42). A cross-sectional study utilizing NHANES data discovered a negative correlation between serum selenium levels and the risk of kidney stones (43). Another study demonstrated an inverse relationship between dietary selenium intake and kidney stone risk, particularly among young men (<50 years) and overweight or obese individuals (BMI ≥ 25.0) (44). Zinc is a crucial component of antioxidant mitochondrial metalloenzymes that exerts its antioxidative effects through binding with metallothionein (45). Additionally, zinc can protect against kidney stones by inhibiting calcium phosphate (CaP) crystallization (46). Studies have indicated that both dietary zinc intake and serum zinc levels are inversely associated with kidney stone prevalence in adults (47). However, a separate study revealed a positive correlation between higher dietary zinc intake and an elevated susceptibility to kidney stone disease (48). The relationship and mechanism between vitamin C intake and kidney stone prevalence remains unclear. On one hand, as a potent antioxidant, vitamin C has the ability to scavenge free radicals and reduce calcium oxalate (CaOx) crystal formation. On the other hand, high vitamin C intake is considered a risk factor for kidney stone formation due to potential increases in urinary oxalate excretion (49). A Prospective cohort analysis revealed that both total vitamin C intake and supplementation were significantly associated with an increased risk of kidney stones in men, while dietary vitamin C intake showed no such association (15). Another study found no significant associations between retinol, β-carotene, vitamins B6, C and E, lycopene and the risk of kidney stones (16). However, when considering the co-existence of vitamin C with other vitamins, contradictory findings were obtained. The combination of vitamin E and vitamin C was reported to effectively reduce urinary calcium oxalate crystals (50). Co-exposure to multivitamins containing vitamin C was found to decrease the risk of kidney stones (51). Although our study also confirmed the protective effects of vitamin C, zinc and selenium on kidney stones, we acknowledge that there are biochemical interactions among antioxidant nutrients which make it challenging to explore and explain their associations with kidney stones using individual components alone (24). Therefore, employing the CDAI as a metric to assess overall antioxidant levels in the diet represents a logical and desirable approach for investigating the association with kidney stone risk.

The outcomes of our study align with prior research demonstrating the protective role of antioxidants against kidney stones (8). Antioxidants can mitigate the risk of kidney stone formation through various mechanisms. These compounds neutralize harmful free radicals in the body, thereby limiting oxidative stress and inflammation, which are recognized as triggers for kidney stone development (52). Elevated peroxidation and diminished thiol levels have the potential to heighten oxalate binding activity, causing harm to renal tubular cells and facilitating nucleation, crystal adhesion, and stone aggregation (14). Similarly, oxidative stress impacts various kidney structures, leading to glomeruli, tubules, and renal vessels, prompting the infiltration of inflammatory cells and the recruitment of proinflammatory cytokines (tumor necrosis factor alpha, TNFα) and transcription factors (nuclear factor kappa, NF-κB).This cascade ultimately leads to an inflammatory phase and subsequent fibrosis that impairs renal function (53). By reducing oxidative stress, antioxidants may help prevent the accumulation of minerals and crystals contributing to stone formation. Antioxidants also have the potential to reduce the body’s production of ROS, which are unstable molecules capable of damaging cells and leading to kidney stones (54). Furthermore, antioxidants can bind with ROS, neutralizing their harmful effects on cells and averting damage. Additionally, antioxidants can stimulate the synthesis of protective substances within the body, such as glutathione, safeguarding cells against ROS-induced harm (55). Moreover, antioxidants can enhance renal health by promoting urine excretion and improving kidney function (56). Collectively, these mechanisms hold promise for minimizing the likelihood of kidney stone occurrence.

In our stratified analysis, we observed a beneficial effect of low-to-moderate alcohol consumption compared to non-alcohol and heavy alcohol consumption on the risk of kidney stones. However, the P for interaction was not statistically significant, indicating that the impact of CDAI on kidney stone risk was not influenced by alcohol intake. Alcohol is known to increase the risk of stones by promoting the formation of uric acid metabolites and causing oxidative stress damage to kidney tissue (57). Nevertheless, the prevailing viewpoint suggests that alcohol can dilute metabolites in blood and urine, inhibit vasopressin secretion, and have a diuretic effect to prevent stone formation (58). A prospective population-based cohort study demonstrated a linear decrease in the risk for kidney stones with increasing alcohol intake; each 200 mL/d increment in alcohol consumption had a hazard ratio (HR) of 0.85 (95% Confidence Interval [CI]: 0.82–0.88) (59). However, excessive alcohol consumption should not be encouraged as it may lead to uncontrolled metabolism and negate the beneficial effects of moderate drinking on cardiovascular health (60). Compared to non-drinkers, individuals consuming 30.0–59.9 g of pure alcohol per day had a reduced risk of kidney stones (HR = 0.79, 95% CI: 0.72–0.87); however, there was no further decrease in risk with higher levels of alcohol consumption (58). Therefore, moderate alcohol consumption may potentially exert a beneficial impact on reducing the risk of kidney stones through a better balance between diuresis and oxidative stress.

As the concentration of oxidative products (including proteins, DNA, and lipids) increases with age, antioxidants can mitigate ROS production and aid in the prevention of age-related diseases such as cardiovascular disease, certain types of cancer, and neurodegenerative diseases (61, 62). However, this does not imply that the elderly derive greater protective benefits against kidney stones from a higher CDAI. Previous studies have demonstrated an inverse association between serum selenium levels and the risk of kidney stone history in the general population. Nevertheless, after conducting age-stratified analysis, only higher serum selenium levels remained significantly negatively associated with kidney stone risk in individuals aged 40–59 years; no significant benefit was observed for those over 60 years old (43). This is consistent with the results of our study. The metabolism of several dietary factors may undergo changes with advancing age，and the relationship between diet and kidney stones may differ among older adults (63). On one hand, intestinal absorption of nutrients that influence stone formation (e.g., calcium) may be diminished in older individuals (64), potentially rendering some antioxidants less effective at inhibiting CaOx crystal formation. On the other hand, aging affects gastrointestinal nutrient absorption function while making older adults particularly susceptible to malnutrition and dysphagia-related issues (65). Consequently, dietary antioxidants might not be fully absorbed or effectively utilized by elderly individuals.

While our study highlights the potential benefits of dietary antioxidants in reducing kidney stone prevalence, it is crucial to note that relying solely on supplements or isolated nutrients is not recommended. A well-balanced diet rich in fruits and vegetables naturally provides a diverse range of antioxidants and other essential nutrients contributing to overall health (66). Additionally, lifestyle factors such as hydration, physical activity, and genetics also play significant roles in kidney stone formation (67). Exploring their interactions with dietary antioxidants should be a focus of future research.

The study demonstrates notable strengths that bolster the robustness and relevance of its findings. A key strength lies in the extensive participant pool drawn from the 2007–2018 NHANES dataset, enhancing both statistical power and the generalizability of conclusions. This wealth of data provides comprehensive insights into the investigated relationship. Additionally, an innovative perspective is introduced by employing CDAI as a novel tool to assess dietary antioxidant intake. This index uniquely considers essential antioxidants, including vitamins A, C, and E, zinc, selenium, and carotenoids, thereby offering a more comprehensive and representative assessment of individuals’ antioxidant consumption patterns. This analytical approach significantly enhances the precision and depth of the investigation.

Acknowledging the study’s design limitations is essential. Firstly, the cross-sectional nature of the study poses a constraint on establishing causality, necessitating further longitudinal studies or interventional trials to establish a more certain causal link. Secondly, evaluating dietary nutrient consumption is based on mean values obtained from two 24-h dietary recalls, which might not completely reflect the daily fluctuations in individuals’ eating habits. Moreover, it is crucial to mention that the outcomes of this study specifically pertain to the population of the United States and cannot be extrapolated to others, thereby requiring further investigation. Lastly, the study’s applicability might be constrained by inherent population-specific or regional factors. Variations in dietary practices, genetic predispositions, and cultural norms could limit the generalizability of the findings beyond the study’s specific participant pool and geographical context.

## 5 Conclusion

The current study reveals a negative correlation between CDAI and incidence of kidney stones in adults, suggesting that higher antioxidant consumption may potentially reduce the chances of kidney stone formation. Nevertheless, the inherent limitations of a cross-sectional design pose challenges in establishing a causal relationship. Therefore, there is a need for prospective research endeavors are warranted to delve deeper into the underlying mechanisms and provide a more comprehensive understanding of this phenomenon.

## Data availability statement

Publicly available datasets were analyzed in this study. This data can be found at: Centers for Disease Control and Prevention (CDC), National Center for Health Statistics (NCHS), National Health and Nutrition Examination Survey (NHANES) database, https://www.cdc.gov/nchs/nhanes/index.htm, NHANES 2007-2018.

## Ethics statement

The studies involving humans were approved by the National Center for Health Statistics (NCHS) Research Ethics Review Board. The patients/participants provided their written informed consent to participate in this study. The studies were conducted in accordance with the local legislation and institutional requirements. The participants provided their written informed consent to participate in this study.

## Author contributions

QD: Conceptualization, Writing – original draft, Writing – review & editing. HH: Data curation, Writing – original draft, Writing – review & editing. SZ: Data curation, Formal analysis, Writing – review & editing. YaW: Data curation, Formal analysis, Writing – review & editing. DL: Data curation, Funding acquisition, Writing – review & editing. LW: Formal analysis, Funding acquisition, Writing – review & editing. YS: Supervision, Writing – review & editing. YoW: Supervision, Writing – review & editing.
