# Supplementary material for: Association between composite dietary antioxidant index and kidney stone prevalence in adults: data from National Health and Nutrition Examination Survey (NHANES, 2007–2018)
Source: Front Nutr. 2024 May 22;11:1389714. doi: 10.3389/fnut.2024.1389714 (PMC11150772; doi:10.3389/fnut.2024.1389714)
Supplement: Supplementary file 1 [file Data_Sheet_1.doc]

Supplementary Material

**Supplementary Table 1.** Baseline characteristics of the general adult population according to quartiles of composite dietary antioxidant index (CDAI) in NHANES 2007–2018.

| Characteristics | Quartiles of composite dietary antioxidant index | | | | P value |
| --- | --- | --- | --- | --- | --- |
|  | Quartile 1 | Quartile 2 | Quartile 3 | Quartile 4 |
| Participants | 7130 | 7131 | 7127 | 7128 |  |
| Age, years | 47.89 (0.34) | 48.60 (0.29) | 47.93 (0.33) | 47.84 (0.36) | 0.111 |
| Sex, % |  |  |  |  | <0.001 |
| Female | 3951 (57.39) | 3568 (50.75) | 3574 (50.63) | 3727 (51.70) |  |
| Male | 3179 (42.61) | 3563 (49.25) | 3553 (49.37) | 3401 (48.30) |  |
| Race/ethnicity, % |  |  |  |  | <0.001 |
| Mexican American | 1025 (8.18) | 1090 (8.66) | 1116 (8.42) | 1021 (7.87) |  |
| Other Hispanic | 796 (6.66) | 747 (5.74) | 746 (5.39) | 676 (5.14) |  |
| Non-Hispanic White | 2775 (63.25) | 2997 (66.71) | 3112 (69.38) | 3104 (69.77) |  |
| Non-Hispanic Black | 1806 (14.39) | 1489 (11.12) | 1369 (9.53) | 1354 (9.06) |  |
| Other race | 728 (7.53) | 808 (7.77) | 784 (7.28) | 973 (8.16) |  |
| Education level, % |  |  |  |  | <0.001 |
| Below high school | 2212 (21.59) | 1809 (16.69) | 1569 (13.53) | 1269 (10.82) |  |
| High school | 1836 (28.22) | 1693 (24.26) | 1537 (21.40) | 1434 (19.22) |  |
| Above high school | 3082 (50.19) | 3629 (59.05) | 4021 (65.07) | 4425 (69.96) |  |
| Family PIR, % |  |  |  |  | <0.001 |
| ≤1.0 | 1937 (20.10) | 1548 (14.58) | 1372 (12.28) | 1263 (11.60) |  |
| 1.1–3.0 | 3219 (40.87) | 3126 (38.27) | 2940 (34.20) | 2744 (31.40) |  |
| >3.0 | 1974 (39.03) | 2457 (47.15) | 2815 (53.52) | 3121 (57.00) |  |
| Smoking status, % |  |  |  |  | <0.001 |
| Never smoker | 3661 (49.57) | 3919 (55.84) | 4084 (57.82) | 4289 (60.18) |  |
| Former smoker | 1593 (22.20) | 1808 (24.91) | 1788 (25.91) | 1764 (26.26) |  |
| Current smoker | 1876 (28.23) | 1404 (19.24) | 1255 (16.27) | 1075 (13.55) |  |
| Drinking status, % |  |  |  |  | <0.001 |
| Nondrinker | 1910 (22.21) | 1612 (18.56) | 1559 (16.77) | 1435 (15.50) |  |
| Low-to-moderate drinker | 4636 (67.91) | 4965 (72.59) | 5006 (73.40) | 5188 (75.90) |  |
| Heavy drinker | 584 (9.88) | 554 (8.85) | 562 (9.83) | 505 (8.60) |  |
| Body mass index, % |  |  |  |  | <0.001 |
| <25.0 kg/m2 | 1951 (28.27) | 1869 (26.94) | 1936 (28.60) | 2230 (32.15) |  |
| 25.0-29.9 kg/m2 | 2278 (31.86) | 2404 (32.84) | 2411 (33.86) | 2310 (32.85) |  |
| >29.9 kg/m2 | 2901 (39.87) | 2858 (40.22) | 2780 (37.54) | 2588 (35.00) |  |
| Physical activity, % |  |  |  |  | <0.001 |
| Inactive | 2292 (26.89) | 2062 (24.12) | 1722 (20.49) | 1594 (18.80) |  |
| Insufficiently active | 2182 (32.66) | 2215 (31.91) | 2321 (33.33) | 2225 (30.78) |  |
| Active | 2656 (40.45) | 2854 (43.97) | 3084 (46.18) | 3309 (50.41) |  |
| Total energy intakes, kcal/day | |  |  |  | <0.001 |
| Quartile 1 | 4195 (56.18) | 1721 (21.97) | 801 (10.29) | 418 (5.12) |  |
| Quartile 2 | 1876 (27.27) | 2439 (33.57) | 1762 (23.90) | 1057 (14.77) |  |
| Quartile 3 | 827 (13.01) | 1997 (29.18) | 2294 (32.40) | 2002 (27.59) |  |
| Quartile 4 | 232 (3.54) | 974 (15.28) | 2270 (33.40) | 3651 (52.53) |  |
| Serum calcium, mmol/L | 2.35 (0.00) | 2.35 (0.00) | 2.35 (0.00) | 2.35 (0.00) | 0.302 |
| eGFR, ml/min/1.73 m2 | 93.22 (0.41) | 93.17 (0.41) | 94.31 (0.42) | 94.43 (0.46) | 0.010 |
| Self-reported hypertension, % | |  |  |  | <0.001 |
| No | 4286 (65.37) | 4391 (65.81) | 4585 (68.72) | 4670 (69.19) |  |
| Yes | 2844 (34.63) | 2740 (34.19) | 2542 (31.28) | 2458 (30.81) |  |
| Self-reported diabetes, % | |  |  |  | 0.005 |
| No | 6036 (88.59) | 6085 (89.17) | 6229 (90.63) | 6275 (90.81) |  |
| Yes | 1094 (11.41) | 1046 (10.83) | 898 (9.37) | 853 (9.19) |  |
| Supplement use, % |  |  |  |  | <0.001 |
| No | 4053 (54.61) | 3563 (47.39) | 3343 (44.23) | 3018 (38.98) |  |
| Yes | 3077 (45.39) | 3568 (52.61) | 3784 (55.77) | 4110 (61.02) |  |
| Self-reported kidney stones, % |  |  |  |  | 0.032 |
| No | 6396 (89.26) | 6391 (89.04) | 6463 (90.23) | 6518 (90.90) |  |
| Yes | 734 (10.74) | 740 (10.96) | 664 (9.77) | 610 (9.10) |  |

Abbreviations: PIR, poverty income ratio; eGFR, estimated glomerular filtration rate; CDAI, composite dietary antioxidant index. Normally distributed continuous variables are described as means ± SEs, and continuous variables without a normal distribution are presented as medians [interquartile ranges]. Sampling weights were applied for calculation of demographic descriptive statistics; N reflect the study sample while percentages reflect the survey-weighted data.


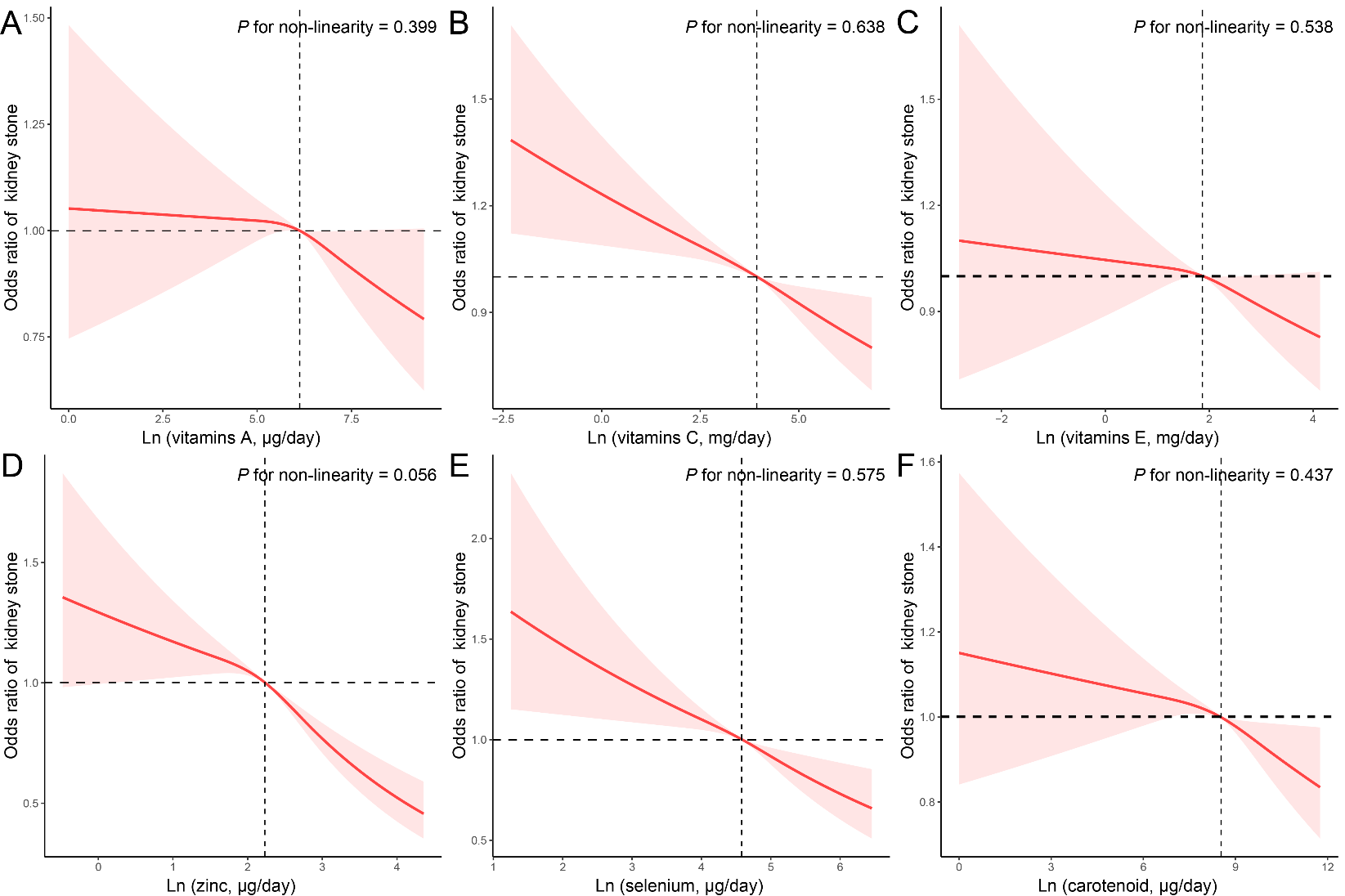


**Supplementary Figure 1.** The exposure-response association of the dietary antioxidant micronutrients (including vitamins A [A], vitamins C [B], vitamins E [C], zinc [D], selenium [E], and carotenoid [F]) with the prevalence of kidney stone by restricted cubic spline (RCS). Analyses were adjusted for covariates age (continuous), sex (male or female), race (Mexican American, Other Hispanic, Non-Hispanic White, Non-Hispanic Black or Other), education level (below high school, high school, or above high school), family income-to-poverty ratio (≤1.0, 1.1–3.0, or >3.0), smoking status (never smoker, former smoker, or current smoker), drinking status (nondrinker, low-to-moderate drinker, or heavy drinker), BMI (<25.0, 25.0-29.9, or >29.9), energy intake levels (in quartiles), physical activity (inactive, insufficiently active, or active), serum calcium (continuous), eGFR (continuous), hypertension (yes or no), diabetes (yes or no), and supplement use (yes or no).

**Supplementary Table 2.** ORs (95% CIs) of the prevalence of kidney stone according to quartiles of composite dietary antioxidant index (CDAI) after excluding participants who had some comorbidities at baseline among adults in NHANES 2007–2018 (n=22,959). *

|  | Crude |  | Model 1 |  | Model 2 |
| --- | --- | --- | --- | --- | --- |
|  | OR (95% CI) |  | OR (95% CI) |  | OR (95% CI) |
| Continuous CDAI | 0.970 (0.952-0.989) |  | 0.966 (0.947-0.985) |  | 0.960 (0.937-0.984) |
| P value | 0.002 |  | <0.001 |  | 0.001 |
| Quartiles of CDAI |  |  |  |  |  |
| Quartile 1 | 1 [Reference] |  | 1 [Reference] |  | 1 [Reference] |
| Quartile 2 | 1.003 (0.835-1.204) |  | 0.951 (0.784-1.154) |  | 0.903 (0.726-1.124) |
| Quartile 3 | 0.880 (0.715-1.082) |  | 0.831 (0.672-1.029) |  | 0.787 (0.622-0.997) |
| Quartile 4 | 0.823 (0.682-0.992) |  | 0.780 (0.643-0.948) |  | 0.740 (0.589-0.928) |
| P for trend | 0.018 |  | 0.006 |  | 0.004 |

Abbreviations: OR, odds ratio; CI, confidence interval; Model 1 was adjusted for age (continuous), sex (male or female), and race (Mexican American, Other Hispanic, Non-Hispanic White, Non-Hispanic Black or Other); Model 2 was adjusted for Model 1 plus education level (below high school, high school, or above high school), family income-to-poverty ratio (≤1.0, 1.1–3.0, or >3.0), smoking status (never smoker, former smoker, or current smoker), drinking status (nondrinker, low-to-moderate drinker, or heavy drinker), BMI (<25.0, 25.0-29.9, or >29.9), energy intake levels (in quartiles), physical activity (inactive, insufficiently active, or active), serum calcium (continuous), eGFR (continuous), hypertension (yes or no), diabetes (yes or no), and supplement use (yes or no).

* Of the 28,516 participants in NHANES 2007-2018 analyzed in this study, 5,557 participants with a history of these comorbidities at baseline were excluded, including cancer (n=2,883), thyroid disease (n=2,264), stroke (n=1,132), and end-stage renal disease (n=100).

**Supplementary Table 3.** ORs (95% CIs) of the prevalence of kidney stone according to quartiles of composite dietary antioxidant index (CDAI) after excluding participants who had fatty liver history at baseline among adults in NHANES 2007–2018 (n=8,084) *.

|  | Crude |  | Model 1 |  | Model 2 |
| --- | --- | --- | --- | --- | --- |
|  | OR (95% CI) |  | OR (95% CI) |  | OR (95% CI) |
| Continuous CDAI | 0.968 (0.934-1.004) |  | 0.964 (0.928-1.002) |  | 0.954 (0.908-1.002) |
| P value | 0.078 |  | 0.060 |  | 0.061 |
| Quartiles of CDAI |  |  |  |  |  |
| Quartile 1 | 1 [Reference] |  | 1 [Reference] |  | 1 [Reference] |
| Quartile 2 | 1.029 (0.793-1.335) |  | 0.976 (0.748-1.274) |  | 0.915 (0.693-1.209) |
| Quartile 3 | 0.877 (0.633-1.214) |  | 0.835 (0.600-1.160) |  | 0.769 (0.526-1.124) |
| Quartile 4 | 0.754 (0.562-0.999) |  | 0.715 (0.525-0.974) |  | 0.630 (0.436-0.911) |
| P for trend | 0.035 |  | 0.022 |  | 0.012 |

Abbreviations: OR, odds ratio; CI, confidence interval; Model 1 was adjusted for age (continuous), sex (male or female), and race (Mexican American, Other Hispanic, Non-Hispanic White, Non-Hispanic Black or Other); Model 2 was adjusted for Model 1 plus education level (below high school, high school, or above high school), family income-to-poverty ratio (≤1.0, 1.1–3.0, or >3.0), smoking status (never smoker, former smoker, or current smoker), drinking status (nondrinker, low-to-moderate drinker, or heavy drinker), BMI (<25.0, 25.0-29.9, or >29.9), energy intake levels (in quartiles), physical activity (inactive, insufficiently active, or active), serum calcium (continuous), eGFR (continuous), hypertension (yes or no), diabetes (yes or no), and supplement use (yes or no).

* There were 21,431 missing values for participants with fatty liver index (FLI). Fatty liver was defined as FLI ≥ 30 (n=4,516).

**Supplementary Table 4.** ORs (95% CIs) of the prevalence of kidney stone according to quartiles of composite dietary antioxidant index (CDAI) after excluding participants who had a history of corticosteroid medication at baseline among adults in NHANES 2007–2018 (n=27,775).

|  | Crude |  | Model 1 |  | Model 2 |
| --- | --- | --- | --- | --- | --- |
|  | OR (95% CI) |  | OR (95% CI) |  | OR (95% CI) |
| Continuous CDAI | 0.971 (0.955-0.987) |  | 0.967 (0.950-0.984) |  | 0.966 (0.947-0.986) |
| P value | <0.001 |  | <0.001 |  | 0.001 |
| Quartiles of CDAI |  |  |  |  |  |
| Quartile 1 | 1 [Reference] |  | 1 [Reference] |  | 1 [Reference] |
| Quartile 2 | 1.026 (0.876-1.202) |  | 0.966 (0.818-1.140) |  | 0.930 (0.776-1.115) |
| Quartile 3 | 0.891 (0.750-1.058) |  | 0.839 (0.703-1.003) |  | 0.814 (0.673-0.986) |
| Quartile 4 | 0.800 (0.670-0.956) |  | 0.755 (0.628-0.909) |  | 0.736 (0.597-0.907) |
| P for trend | 0.003 |  | <0.001 |  | 0.001 |

Abbreviations: OR, odds ratio; CI, confidence interval; Model 1 was adjusted for age (continuous), sex (male or female), and race (Mexican American, Other Hispanic, Non-Hispanic White, Non-Hispanic Black or Other); Model 2 was adjusted for Model 1 plus education level (below high school, high school, or above high school), family income-to-poverty ratio (≤1.0, 1.1–3.0, or >3.0), smoking status (never smoker, former smoker, or current smoker), drinking status (nondrinker, low-to-moderate drinker, or heavy drinker), BMI (<25.0, 25.0-29.9, or >29.9), energy intake levels (in quartiles), physical activity (inactive, insufficiently active, or active), serum calcium (continuous), eGFR (continuous), hypertension (yes or no), diabetes (yes or no), and supplement use (yes or no).


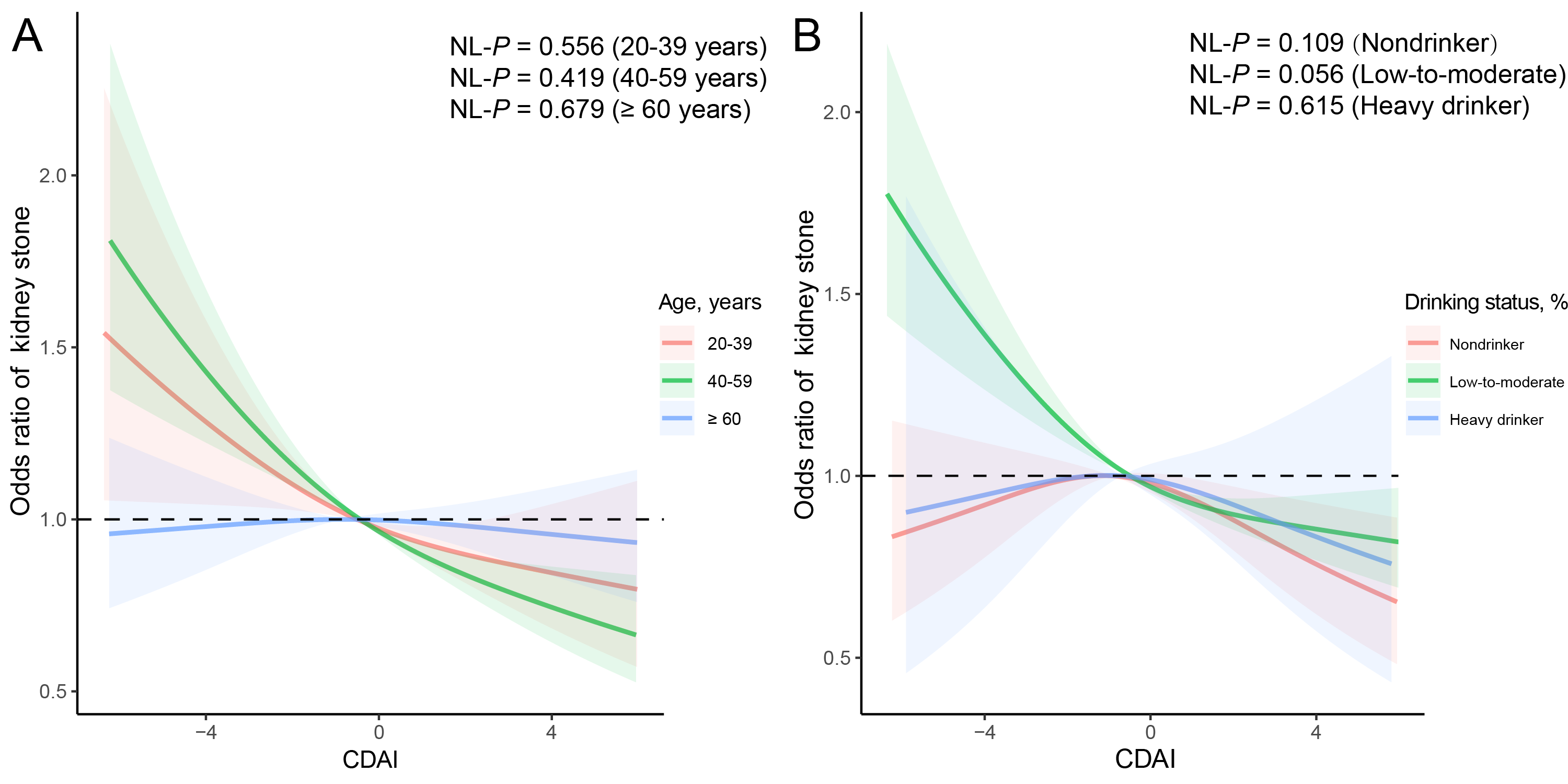


**Supplementary Figure 2.** The association of CDAI with the prevalence of kidney stone in stratifying alcohol consumption and age by restricted cubic spline (RCS).
